# Expansion and characterization of human limbus-derived stromal/mesenchymal stem cells in xeno-free medium for therapeutic applications

**DOI:** 10.1186/s13287-023-03299-3

**Published:** 2023-04-15

**Authors:** Abhishek Sahoo, Mukesh Damala, Jilu Jaffet, Deeksha Prasad, Sayan Basu, Vivek Singh

**Affiliations:** 1 Centre for Ocular Regeneration, Prof. Brien Holden Eye Research Centre, L V Prasad Eye Institute, Hyderabad, Telangana India; 2 Department of Animal Biology, School of Life Sciences, University of Hyderabad, Hyderabad, Telangana India; 3 Manipal Academy of Higher Education, Manipal, Karnataka India

**Keywords:** hLMSCs, Xeno-free media, Corneal scarring, Regenerative medicine, Therapeutic applications

## Abstract

**Background:**

Mesenchymal stem cells (MSCs) have been proven to prevent and clear corneal scarring and limbal stem cell deficiency. However, using animal-derived serum in a culture medium raises the ethical and regulatory bar. This study aims to expand and characterize human limbus-derived stromal/mesenchymal stem cells (hLMSCs) for the first time in vitro in the xeno-free medium.

**Methods:**

Limbal tissue was obtained from therapeutic grade corneoscleral rims and subjected to explant culture till tertiary passage in media with and without serum (STEM MACS XF; SM), to obtain pure hLMSCs. Population doubling time, cell proliferation, expression of phenotypic markers, tri-lineage differentiation, colony-forming potential and gene expression analysis were carried out to assess the retention of phenotypic and genotypic characteristics of hLMSCs.

**Results:**

The serum-free medium supported the growth of hLMSCs, retaining similar morphology but a significantly lower doubling time of 23 h (*p < 0.01) compared to the control medium. FACS analysis demonstrated ≥ 90% hLMSCs were positive for *CD90*^+^, *CD73*^+^, *CD105*^+^, and ≤ 6% were positive for *CD45*^−^, *CD34*^−^ *and* *HLA-DR*^−^*.* Immunofluorescence analysis confirmed similar expression of *Pax6*^+^, *COL* *IV*^+^, *ABCG2*^+^, *ABCB5*^+^, *VIM*^+^, *CD90*^+^, *CD105*^+^, *CD73*^+^, *HLA-DR*^−^ *and* *CD45*^−^, *αSMA*^−^ in both the media. Tri-lineage differentiation potential and gene expression of hLMSCs were retained similarly to that of the control medium.

**Conclusion:**

The findings of this study demonstrate successful isolation, characterization and culture optimization of hLMSCs for the first time in vitro in a serum-free environment. This will help in the future pre-clinical and clinical applications of MSCs in translational research.

**Supplementary Information:**

The online version contains supplementary material available at 10.1186/s13287-023-03299-3.

## Key findings

- This study successfully optimizes the growth and expansion of hLMSCs in a serum-free environment using commercially available SM medium, retaining their spindle-shaped morphology till higher generations (P8).
- hLMSCs cultured in SM maintained significantly lower population doubling time, tri-lineage differentiation potential and significantly higher colony-forming ability than the control medium. Immunofluorescence and FACS analysis showed expression of MSC markers and absence of expression of myo-fibroblastic marker (αSMA), and haematopoietic markers (CD45, CD34 and HLA-DR).
- The qPCR data suggested similar expression of MSC phenotypic markers, wound healing markers and inflammatory markers identical to that of the control medium.

**Supplementary Information:**

The online version contains supplementary material available at 10.1186/s13287-023-03299-3.

## Background

MSCs originate from mesoderm during embryo development, possess fibroblastic and spindle shape morphology, adhere to the plastic surface, and positively express phenotypic cell surface markers CD105 (endoglin-recognized by SH2), CD90 (Thy-1), CD73 (ecto 5′ nucleotidase -recognized by SH3 and SH4), CD106, CD166, COL-I, COL-III and is negative for hematopoietic markers CD34 (primitive hematopoietic progenitors and endothelial cell marker), CD45 (pan-leukocyte marker), CD19, HLA-DR, CD14 & 11b (monocytes and macrophages) and α-SMA [1, 2]. Another specific criterion of MSC is to differentiate into Adipocytes (Oil Red O staining), Osteocytes (Alizarin Red staining for Ca^2+^ deposits) and Chondrocytes (Alcian Blue staining for collagen type II and GAGs) upon induction. In vitro culture and expansion of these cells are of utmost necessity for cell-based therapies. Isolation and culture of MSC date back to the 1970s, when Friedenstein et al. [3] first described them, and since then, various methods and protocols have been devised to cultivate these cells. The field of regenerative medicine has paved the way for MSCs to become an emerging player owing to its translational applications [4]. Cell-based therapies have become immensely popular in regenerative medicine to repair or regenerate a tissue through stem cell transplantation [5]. Currently, 352 clinical trials have been completed, and 339 are ongoing using MSCs as cellular therapy; (https://clinicaltrials.gov/ct2/home); 26/11/2022.

MSCs have been isolated from various tissues in the body like bone marrow (BM), umbilical cord (UC), adipose tissue (AD), dental pulp, corneal and limbal stroma [6, 7]. The cornea is a transparent and avascular connective tissue on the anterior eye, forming a barrier between the eye and the outside world [8]. It is divided into five layers depending on function and anatomy. The outermost layer is corneal epithelium followed by compactly arranged stroma consisting predominantly of type I and V collagen fibrils, separated by Bowman’s membrane. Inner to stroma lies Descemet’s membrane preceding a monolayer of cuboidal cells known as corneal endothelium. To perceive vision, the cornea refracts two-thirds of the total light onto the retina [7]. As evident from the literature, corneal stroma houses a specific cell population satisfying the MSC criterion known as corneal stromal stem cells (CSSC) that eventually differentiate into stromal keratocytes [7–11]. These stromal keratocytes differentiate into fibroblastic scar tissue upon corneal injury, thus opacifying the transparent cornea and leading to vision deterioration [12–19]. The most common and accepted treatment owing to this visual impairment is corneal transplantation (keratoplasty); however, the availability of suitable donor corneas falls behind the demand. Furthermore, post-operative complications and immune rejection of corneal allografts add to the disadvantages [20, 21]. To cater to the above needs, various therapeutic alternatives to keratoplasty, like stem cell therapy, 3D printed corneas, and bio-mimetic hydrogels, are currently being explored [11, 22].

Mesenchymal stem cells (MSCs) have become promising candidates for various biologically engineered therapeutic applications. The presence of multipotent mesenchymal stem cells in corneal stroma has paved the way for cellular therapy against disorders like limbal stem cell deficiency (LSCD). The efficacy of MSCs as a therapeutic agent in restoring corneal transparency has been confirmed in previous studies. Moreover, MSCs being immunosuppressant, post-operative complications like immune rejection and inflammation can be ruled out [23–25]. The corneoscleral junction of the human eye, known as limbus, houses mesenchymal stromal cells in finger-like projections called palisades of Vogt. These cells can be cultured from limbal biopsy, expanded using an appropriate growth medium and used for treating LSCD, corneal burns, scars and various ocular surface injuries [19]. MSCs isolated from human umbilical cord have been shown to restore corneal transparency in Lumican knockout mice, thus confirming that stem cells from different organs aid in corneal regeneration  [26]. In humans, MSCs from other tissues also have been reported to cure corneal scarring and restore transparency [27–30].

As the evolvement of science is marching forward, the procedure to develop an optimal culturing condition is also being looked for alternative ways to cultivate MSCs [31, 32]. For half a century, FBS has been acting as a supplement to the basal media in most of the studies and even in clinical trials [33–45]. FBS is an ill-defined pool of macro- and micro-molecules required for the growth and sustainability of cells. Being of xenogeneic origin and with lot-to-lot variance, FBS is prone to cause zoonotic diseases like anthrax, Q fever, and Creutzfeldt–Jakob Disease (CJD). Bovine Spongiform Encephalopathies and their relation to the new variant of CJD can also be caused due to the presence of harmful pathogens like unknown viruses, bacteria, prions and endotoxins in FBS [46]. Cells cultured in FBS are prone to be contaminated with mycoplasma which is unnoticeable and can easily pass through 0.22µ filters [47, 48]. Furthermore, slaughtering a bovine foetus for serum extraction is inhumane and questions the ethical issues in many countries [49]. Large-scale production of serum is uneconomical, pointing to the high cost of feeding, maintenance and infrastructure for bovine rearing [50].

To tackle these limitations, the regulatory authorities have emphasized looking for a serum-free medium to delineate a standardized paradigm that can preserve the therapeutic potential of MSCs [5]. Serum-free or xeno-free medium formulations are chemically defined mediums that need strict optimization and characterization based on specific cell types [51]. Recently various industry groups like RoosterBio, Inc., MD, USA (RoosterNourish-MSC XF); Miltenyi Biotec, Germany (STEM MACS XF); Merck, USA (PLTMax Human Platelet Lysate); R&D Systems, USA (StemXVivo Serum free Human Mesenchymal Stem Cell Expansion media) and many more are focusing on optimizing FBS free culture medium [52]. Several research groups are looking into preparing in-house xeno-free medium for the growth and expansion of MSCs [39, 53–58].

This study aims to optimize and characterize SM to expand hLMSCs to higher passages and check their suitability for therapeutic use. This chemically defined xeno-free medium is formulated by Miltenyi Biotec and manufactured in compliance with cGMP regulations. This is a patented proprietary chemically defined medium, (cat no.- 130-104-182) so disclosure of individual components is limited to the manufacturer only. The details about SM can be accessed from; (https://www.miltenyibiotec.com/US-en/products/stemmacs-msc-expansion-media-kit-xf-human.html#gref). SM had previously been reported to support the growth and expansion of adult mesenchymal stem cells, [4] which prompted us to test it in human limbal stromal cells.

## Materials and methods

### Isolation and culture of hLMSCs

The complete limbal rim was dissected from the therapeutic grade and biologically tested cadaveric corneas obtained from Ramayamma International Eye Bank (RIEB) (http://www.lvpei.org/services/eyebank) with proper documentation. This study was approved by the Institutional Review Board (IRB) of the LV Prasad Eye Institute (Ethics Ref. No. LEC-05-18-081) and Institutional Committee for Stem Cell Research (IC-SCR Ref No 08-18-002) and followed the tenets of the declaration of Helsinki. Briefly, the corneoscleral rim was first washed with 2× [vol/vol] Antibiotic–Antimycotic solution (15240062, Thermo Fisher, USA) in sterile filtered Phosphate Buffer Saline (PBS) (D5652-10L, Sigma-Aldrich). Iris and tissue debris, if any, were cleaned with the help of a scalpel blade (15 no.) in 1X PBS. Carefully, a 360° limbal rim of about 1–2 mm diameter was dissected from the corneoscleral rim and cut into small fragments of 1–2 mm length. The fragmented tissue was minced with the help of curved scissors in 1 mL of sterile-filtered DMEM/F12 medium (D0547-10X1L, Sigma-Aldrich). The minced tissue was subjected to collagenase digestion by adding 200 IU of reconstituted collagenase IV (17104019, Thermo Fisher, USA) in HBSS buffer (14025092, Thermo Fisher, USA) and incubated for 16 h at 37 °C and 5% CO_2_.

Post-digestion, 1 mL of DMEM/F12 complete medium fortified with 2% of foetal bovine serum (SH30396.03, Cytiva Life Sciences), 1% [vol/vol] Antibiotic–Antimycotic, 10 ng/mL epidermal growth factor (PHG0311L, Thermo Fisher, USA) and 5 µg/mL insulin (12585014, Thermo Fisher, USA), was added. The solution was spun down at 1000 rpm for 3 min at 25 °C. The pellet was then washed twice with 1X PBS. 2 mL of complete medium was added to the final pellet, mixed well and kept for growth in a T25 flask at 37 °C and 5% CO_2_ with media changed every 3rd day. This served as a control for this study.

For culturing the cells in SM (130-104-182, Miltenyi Biotec), post-enzymatic digestion, 1 mL of SM medium was added and centrifuged at 1000 rpm for 3 min, following two PBS washes. 2 mL of SM medium was added to the pellet and was kept in culture maintained at 37 °C, and 5% CO_2_ with media changed every 3rd day.

As this is a primary culture, we obtain a mixed population of limbal epithelial and stromal cells in the first two passages. Pure population of MSCs are obtained at third passage of the culture. Hence, this pure population of MSCs (henceforth referred to as P3 hLMSCs) were used for all the characterization experiments post-viability quantification using 0.4% Trypan Blue (15250061, Thermo Fisher, USA).

### Population doubling time (PDT) and cumulative population doublings (CPD)

To look into the growth kinetics, hLMSCs cultured in both media were used. Briefly, 1 × 10^4^ cells were seeded in triplicates in a 48-well plate and harvested upon 80–90% confluency, and the growth duration was noted. The total viable cell number was recorded, and Population Doubling Time (PDT) was calculated from P3 to P8 using the formula below from https://www.doubling-time.com/compute.php.

$${\text{Doubling}}\,{\text{Time}} = \frac{{{\text{Duration}}*{\text{log}}\,(2)}}{{{\text{Log}}({\text{Final}}\,{\text{Concentration}}){-}{\text{log}}({\text{Initial}}\,{\text{Concentration}})}}$$

Cumulative Population Doublings (CPD) were calculated using the formula (Initial PDT + 3.322*(log (Cell no. at confluency) − log (seeding cell no.)).

### Relative viability assay using MTT

5000 cells/cm^2^ of hLMSCs were seeded in a 48-well plate in triplicates separately in SM and serum-based medium and cultured for 24, 48, 72, 96 and 120 h at 37 °C and 5% CO_2_ in a humidified incubator. Post 24 h of seeding (termed as T_0_), spent media was removed, and 200 µL of 2 mg/mL of MTT (M6494, Thermo Fisher, USA) dissolved in DMEM/F12 devoid of growth supplements and FBS was added preceding a PBS wash and incubated for 3 h at 37 °C and 5% CO_2_. Post-incubation, 200 µL DMSO (D2650, Sigma-Aldrich, USA) was added following 10 min incubation to solubilize the formazan crystals. 100 µL of supernatant was transferred to a transparent bottom 96-well plate, and absorbance was taken in triplicates at 570 nm in a UV–Vis spectrophotometer against DMSO as blank. The same steps were repeated for *T*_24_, *T*_48_, *T*_72_, *T*_96_, and *T*_120_ h.

### Immunophenotypic markers expression by immunofluorescence and flow cytometry

To assess the expressivity of MSC phenotypic markers, hLMSCs grown in both the medium were subjected to immunofluorescence (IF) and FACS analysis. In brief, for IF, cells were seeded on coverslips placed in 12-well plates at a density of 5000 cells/cm^2^. Upon 60–70% confluency, the cells were washed with 1× PBS, fixed in 4% paraformaldehyde for 20 min, followed by two PBS washes for 5 min each. The fixed cells were permeabilized using 0.5% triton-X for 5 min, following three PBS washes of 5 min each. Thereafter, hLMSCs were incubated for 45 min at room temperature in 2.5% BSA in PBS (blocking buffer) to restrict non-specific interactions. Post-blocking, the cells were incubated with primary antibody dissolved in 1% BSA solution and kept at 4 °C overnight. The antibody panel consisted of CD105 (1:200, ab156756, Abcam, UK), CD90 (1:200, ab181469, Abcam, UK), ABCG2 (1:200, ab229193, Abcam, UK), ABCB5 (1:200, ab140667, Abcam, UK), COLIV (1:200, ab6586, Abcam, UK), CD73 (1:100, 13160S, Cell Signalling Technology, USA), p63-α (1:100, ab124762, Abcam, UK), and Vimentin (1:100, sc-6260, Santa Cruz Biotechnology, USA) as positive markers of the mesenchymal phenotype, Pax6 (1:200, ab195045, Abcam, UK) as positive markers of the human limbal stem cell phenotype; HLA-DR (1:100, ab92511, Abcam, UK), and CD45 (1:100, ab154885, Abcam, UK), αSMA (1:100, MA5-11547, Invitrogen, USA), as negative markers for mesenchymal stem cells origin according to the guidelines of ISCT. To nullify the presence of epithelial phenotype in P3 hLMSCs, IF was carried out using epithelial markers, CK3/2P (1:100, sc-80000, Santa Cruz Biotechnology, USA), CK14 (1:100, sc-53253, Santa Cruz Biotechnology, USA) and CK15 (1:100, sc-47697, Santa Cruz Biotechnology, USA); (Additional file 1: figure S1).

Post-incubation, cells were washed twice for 5 min each with PBS, and 1:400 dilution of secondary antibodies in 1% BSA was added, followed by incubation at R.T. Secondary antibodies included anti-mouse Alexa Flour 488 (A11001, Thermo Fisher, USA) and anti-rabbit Alexa Flour 488 (A11008, Thermo Fisher, USA). After 45 min of incubation, cells were washed thrice with PBS to remove the background stain and mounted using a Mounting Medium with DAPI - Aqueous, Fluoroshield (ab104139, Abcam, USA). Fluorescent images of mounted cells were captured with Zeiss LSM 880(Carl Zeiss AG, Germany).

FACS analysis of hLMSCs cultured in SM and control medium was carried out to quantify the expression of phenotypic markers. P3 hLMSCs were trypsinized upon 70–80% confluency, and around 1 × 10^5^ cells were added to 1.5 mL vials. The cell suspension was spun down at 400 g for 5 min following two PBS washes. Conjugated antibodies were dissolved in 2% FBS in PBS (blocking buffer) in 1:100 dilution following incubation for 20 min in the dark at room temperature. The phenotypic markers analysed were CD105^+^ (B76299), CD90^+^ (B36121), CD73^+^ (B68176), CD45^−^ (A07783), CD34^−^ (IMI870), HLA-DR^−^ (B36291), all from Beckman Coulter (Brea, CA, USA). Post-incubation, cells were washed twice in 1X PBS, and the pellet was resuspended in 200 µL of PBS. The stained cells were analysed using CytoFLEX flow cytometer (Beckman Coulter, CA, USA), and the data analysis was carried out using CytExpert software (Beckman Coulter, CA, USA).

### Tri-lineage differentiation

The potential of both the medium to support tri-lineage differentiation was evaluated using MesenCult™ Adipogenic (05412), Osteogenic (05465), and Chondrogenic (05455) differentiation kit (Stem Cell Technologies, USA). Briefly, 5000 cells/cm^2^ of hLMSCs were seeded in triplicates in 24 well plate. Upon 60–70% confluency, the cells were induced to differentiate for 21 days. The media was changed every 3rd day, and the plate was periodically observed for differentiation. Post 21 days, the cells were fixed with 4% paraformaldehyde for 10 min following two PBS washes. Staining was carried out using alizarin red for osteogenic differentiation, Alcian blue for chondrogenic differentiation, and oil red O for adipogenic differentiation for 10 min, 1 h and 20 min, respectively. Three washes with Milli-Q water were given to drain out excessive stains, and differentiated cells were imaged in PBS under a bright field microscope.

To quantify the extent of differentiation in SM and control media, stain from individual wells was eluted using various dye elution techniques [59–61] and the intensity was measured using a UV–Vis spectrophotometer (SpectraMax M3, Molecular Devices, California, USA).

### Colony forming unit (CFU) assay

hLMSCs (1000 cells) were seeded in 70 mm tissue culture Petri dishes in SM and control medium for 14 days at 37 °C and 5% CO_2_, replenishing media every 3–4 days. Post day 14, the colonies were fixed with ice-cold methanol for 10 min at 4ºC following incubation with 0.5% crystal violet for 10 min. The dish was washed 2–3 times with tap water and Milli-Q water to remove the excess stain. The no. of visible colonies with a size more than 2 mm was counted manually. A histogram was plotted between means of the total no. of colonies observed in both media.

### In vitro wound-healing assay

hLMSCs were cultured in serum-based and SM medium in a six-well plate till 80%-90% confluency. The monolayer of cells was scratched using a sterile 200 µL pipette, following a PBS wash to remove the floating cells. Images were taken immediately after scratching and after 12, 24, 36 and 48 h, respectively, to look for the cell migration for wound healing. The decrease in the wounded area was measured using ImageJ software [62], to determine the healing potency.

### Gene expression analysis using real-time PCR

Total RNA was isolated after resuspending in RNAiso Plus (9108/9109, TAKARA). RNA isolation was done using the traditional phenol–chloroform method following quantification using Nanodrop. cDNA was synthesized using SuperScript™ III First-Strand Synthesis System (18080051, Thermo Scientific™) by taking an equal RNA concentration in all the samples. Maxima SYBR Green/ROX qPCR Master Mix- 2X (F416L, Thermo Scientific™) kit was used for gene expression analysis on QuantStudio™ 3 Real-Time PCR System (A28567, Applied Biosystems™) using gene-specific primers as listed in Table 1. GAPDH was taken as the reference gene. RNA from human cadaveric limbal tissue was used as the control for this assay. The relative fold change of various genes was calculated using the 2^−(ΔΔct)^ method. The graph was plotted on a logarithmic scale, taking relative fold change on the *Y* axis and genes on the *X* axis.

**Table 1:** List of primers used in this study for gene expression analysis

Sl. no	Genes	Primer sequence		Size (bp)
1	LUMICAN	Fwd	GCACAATCGGCTGAAAGAGG	228
		Rev	TCAGCCAGTTCGTTGTGAGA	
2	IL10	Fwd	GCTGGAGGACTTTAAGGGTTACCT	109
		Rev	CTTGATGTCTGGGTCTTGGTTCT	
3	IL 6	Fwd	GCGATGGAGTCAGAGGAAACT	218
		Rev	AGTGACTCAGCACTTTGGCA	
4	COL1A1	Fwd	GTCACCCACCGACCAAGAAACC	121
		Rev	AAGTCCAGGCTGTCCAGGGATG	
5	COL5A1	Fwd	TTCAAGCGTGGGAAACTGCT	115
		Rev	GGTAGGTGACGTTCTGGTGG	
6	TGFβ1	Fwd	TACCTGAACCCGTGTTGCTCTC	122
		Rev	GTTGCTGAGGTATCGCCAGGAA	
7	COL3A1	Fwd	TGAAAGGACACAGAGGCTTCG	532
		Rev	GCACCATTCTTACCAGGCTC	
8	P63α	Fwd	ACCTGGAAAACAATGCCCAGA	369
		Rev	GAGGTGGGGTCATCACCTTG	
9	VIM	Fwd	GGACCAGCTAACCAACGACA	178
		Rev	AAGGTCAAGACGTGCCAGAG	
10	CD105	Fwd	CGGTGGTCAATATCCTGTCGAG	109
		Rev	AGGAAGTGTGGGCTGAGGTAGA	
11	CD90	Fwd	AGCATCGCTCTCCTGCTAAC	230
		Rev	CTGGTGAAGTTGGTTCGGGA	
12	CD73	Fwd	GGCTGCTGTATTGCCCTTTG	175
		Rev	TACTCTGTCTCCAGGTTTTCGG	
13	RUNX2	Fwd	CCACTGAACCAAAAAGAAATCCC	129
		Rev	GAAAACAACACATAGCCAAACGC	
14	CD45	Fwd	CTTCAGTGGTCCCATTGTGGTG	107
		Rev	CCACTTTGTTCTCGGCTTCCAG	
15	KERA	Fwd	GACACAGGACTCAACGGTGT	205
		Rev	GTAGGAAAACTGGGTGGGCA	
16	ALDH3A1	Fwd	CAGTTACCGGGAGAGGCTGT	345
		Rev	GTGGCTCCGAGTGGATGTAG	
17	SEMA3A	Fwd	AGACTCACTTGTACGCCTGTG	242
		Rev	CCCAAGAGTTCGGAAGATAGCAA	
18	DCN	Fwd	ATGAAGGCCACTATCATCCTCC	135
		Rev	GTCGCGGTCATCAGGAACTT	
19	COL4A1	Fwd	TGTTGACGGCTTACCTGGAGAC	120
		Rev	GGTAGACCAACTCCAGGCTCTC	
20	PAX6	Fwd	ATAACCTGCCTATGCAACCC	208
		Rev	GGAACTTGAACTGGAACTGAC	
21	IL1β	Fwd	CCTGTCCTGCGTGTTGAAAGA	149
		Rev	GGGAACTGGGCAGACTCAAA	
22	TNFα	Fwd	CCCCAGGGACCTCTCTCTAATC	94
		Rev	GGTTTGCTACAACATGGGCTACA	

### Statistical analysis

All the mean, standard deviation and standard error of mean were calculated in Microsoft Excel, and the graphs were plotted using the GraphPad Prism application (GraphPad Software, SanDiego, USA). Statistical significance was analysed using the student’s *t* test for non-parametric data. *p* < 0.05 were considered significant and represented by *, whereas *p* > 0.05 meant non-significant and is represented as “ns”.

## Results

### Isolation and culture of hLMSCs

**Fig. 1:**
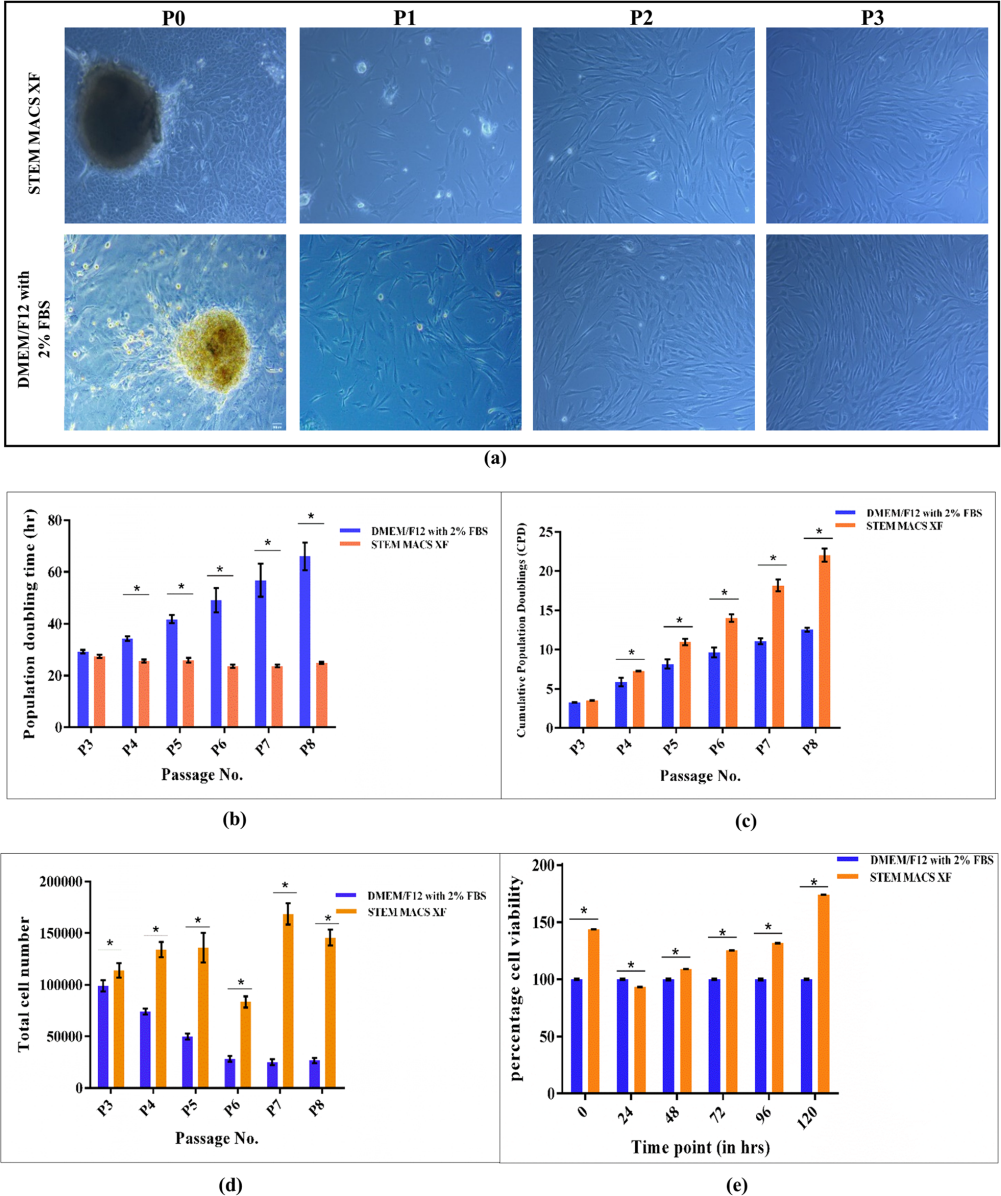
**a** Micrographs of hLMSCs cultured in SM and Control medium, respectively. Cells grown in SM retained spindle-shaped morphology till passage P8, but cells grown in the control medium showed elongated fibroblastic morphology from P4. At P3, hLMSCs had spindle morphology in both the media. Magnification: × 10, Scale: 200 µM; **b** relative Population Doubling Time (PDT) of hLMSCs. hLMSCs cultured in SM displayed lower PDT than in the control medium. PDT was maintained till passage 8 in SM, which increased relatively in the control medium after passage 4. The data are represented as mean ± SD; **c** cumulative population doublings of hLMSCs in control and SM. SM showed comparatively more population doublings than the control medium; **d** bar graph representing total no. of viable cells at subsequent passages in control and SM medium; **e** percentage viability of hLMSCs cultured in SM and control medium was measured by MTT assay. SM cultures displayed cell viability similar to the control medium till 96 h and increased after that. The X and Y axes represent the time point and percentage cell viability, respectively. The percentage viability of cells in the control medium is taken as 100%. Data are expressed as mean ± SD in triplicates; *n* = 3; *p* < 0.01

Serum-free (SM) and control media supported the attachment and growth of cells from limbal explants (*n* = 3). At P0, a mixed population of limbal epithelial and stromal cells were seen (Fig. 1a). The epithelial cells were cuboidal/polygonal in shape, and stromal cells showed spindle morphology. The microscopic images revealed that subsequent passages resulted in a decrease in epithelial cell population and an increase in the number of elongated, spindle-shaped stromal cells. A pure population of hLMSCs (P3 cells) were obtained at the end of 3rd passage; hence, these cells were used for further characterization.

### PDT, CPD and relative viability rate using MTT

At each passage, viable cell count in SM outnumbered cells in the control medium. The total viable cell count at various passages in both the media is represented in Table 2. The population doubling time (25 ± 2 h.) was retained until further passages in SM, whereas it increased to 66 h. in the control medium. The graph was plotted taking PDT on the Y-axis and subsequent passages on the X-axis (Fig. 1b).

**Table 2:** The viable count of hLMSCs cultured in SM and in control medium at different passages

Passage No	Cell count in control medium	Cell count in SM
P3	0.98 × 10^5^ ± 0.054	1.1 × 10^5^ ± 0.068
P4	0.62 × 10^5^ ± 0.028	1.34 × 10^5^ ± 0.074
P5	0.49 × 10^5^ ± 0.028	1.36 × 10^5^ ± 0.143
P6	0.27 × 10^5^ ± 0.027	0.83 × 10^5^ ± 0.054
P7	0.27 × 10^5^ ± 0.031	1.6 × 10^5^ ± 0.10
P8	0.27 × 10^5^ ± 0.026	1.2 × 10^5^ ± 0.076

As Cumulative Population Doublings (CPD) and PDT are inversely proportional to each other, CPD was seen to be increasing significantly in case of SM in comparison with the control medium due to significantly lower PDT of SM (Fig. 1c). Owing to a significant difference in PDT between two media, the relative viable rate was evaluated in both media. To assess the relative viability rate of hLMSCs, an MTT assay was carried out. As evident from the graph (Fig. 1e), cells in serum-free medium (SM) showed significantly higher viability than in the control medium. The data were normalized to that of the control medium, which was taken as 100%. (*p < 0.01, for graphs 1 b–e).

### Phenotypic expression of markers using immunofluorescence and flow cytometry

**Fig. 2:**
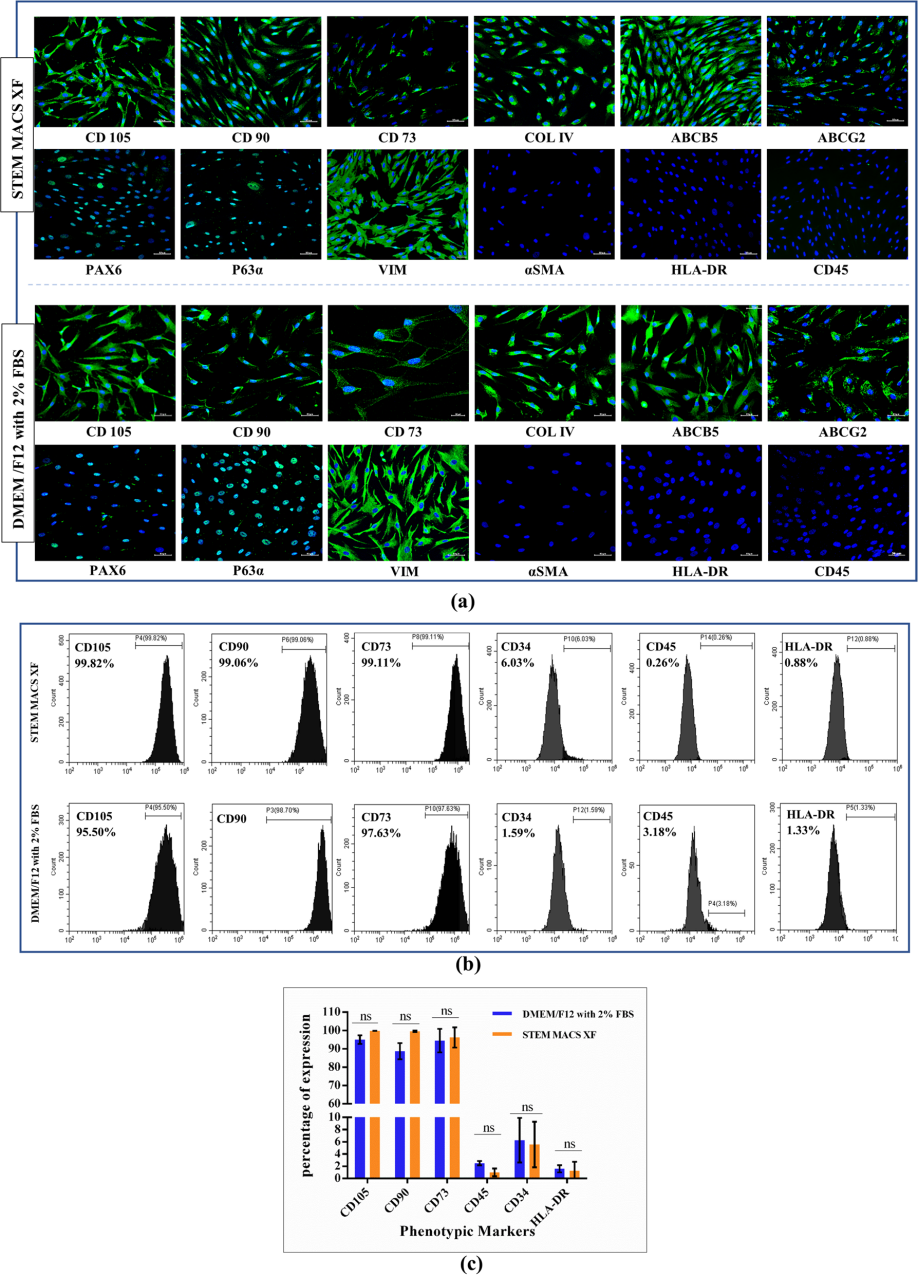
**a** Immunofluorescence analysis showed approximately similar biomarker expression of hLMSCs in SM for ocular surface marker (Pax6^+^), stem-cell biomarkers (ABCG2^+^, P63α^+^, ABCB5^+^) and the mesenchymal biomarkers (VIM^+^, CD90^+^, CD105^+^, CD 34^−^, HLA-DR^−^ and CD45^−^) with respect to the cells in control medium; **b** the expression of MSC markers in hLMSCs grown in both the media was quantified using flow cytometry. More than 97% of cells were positive for CD105, CD90, and CD73, whereas less than 1% showed expression for negative markers CD45 and HLA-DR and approx. 6% of total cells were positive for CD34; **c** graphical representation of flow cytometry data. Blue: DAPI; Scale: 50 µM; Magnification: × 20 (all other micrographs) and 20 µM (CD73 of DMEM/F12 with 2% FBS; × 40 magnification)

The immunophenotype marker expression of hLMSCs cultured in serum-free (SM) and serum-based media (control medium) was evaluated using immunofluorescence (IF) staining and flow cytometry. hLMSCs stained their characteristics phenotype ocular surface marker (Pax6^+^, COL IV^+^), stem cell biomarkers (P63α^+^, ABCG2^+^, ABCB5^+^) and the mesenchymal biomarkers (VIM^+^, CD90^+^, CD105^+^, CD73^+^, HLA-DR^−^, αSM A^−^ and CD45^−^) adapting to serum-free conditions (Fig. 2a).

FACS analysis showed no significant difference in the expression of phenotypic markers of hLMSCs grown in both media. The expression was ≤ 6% of negative MSC markers (CD45, CD34 and HLA-DR) and ≥ 97% expression of positive markers (CD105, CD73, CD90). (Fig. 2b, c).

### Tri-lineage differentiation

To assess the effect of serum-free medium on the tri-lineage differentiation potential of hLMSCs, in vitro differentiation was carried out. Osteogenic differentiation was marked as deep red colour calcium deposits after staining with Alizarin Red. Approximately 80–90% of the total area was stained red in both media showing efficient differentiation. Red fat droplets identified adipogenic differentiation after staining with Oil Red O. Cells grown in SM had clustered droplets, whereas control cells showed individual fat vacuoles. Glycosaminoglycan (GAG) deposits stained with Alcian Blue marked the chondrogenic differentiation.

**Fig. 3:**
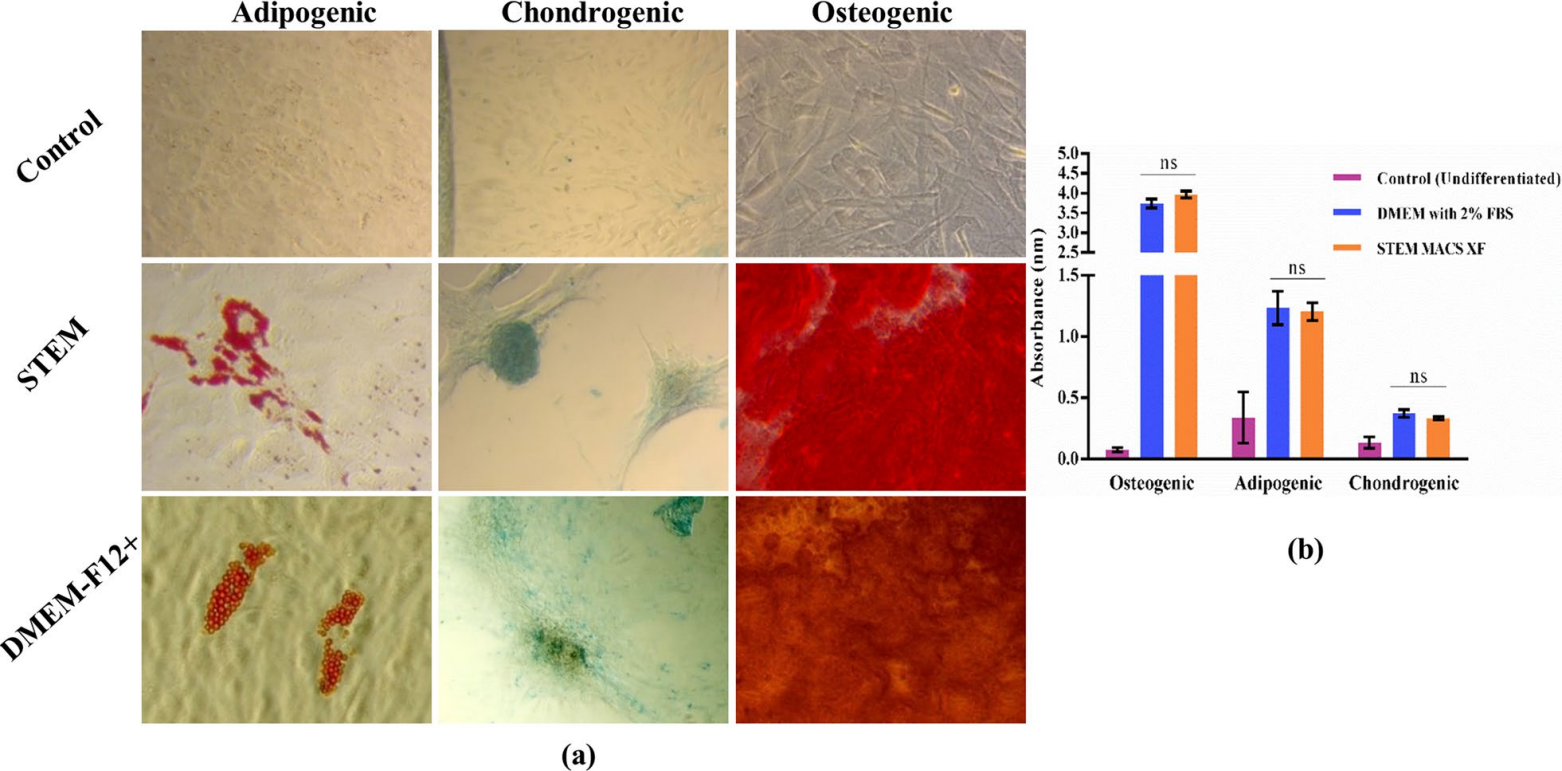
**a** Tri-lineage differentiation potential of hLMSCs cultured in SM and control medium. Control represents undifferentiated cells. Adipogenic differentiation was identified by the formation of oil droplets stained by Oil Red O stain. Chondrogenic differentiation had glycosaminoglycans stained by an acidic stain, Alcian Blue. Osteogenic differentiation was identified as a large number of calcium deposits stained by Alizarin Red stain. hLMSCs cultured in both the medium showed a significant amount of tri-lineage differentiation; **b** graph of quantification of tri-lineage differentiation of hLMSCs into osteocytes, adipocytes and chondrocytes. The respective stains were eluted, and the intensity of colour was measured using a spectrophotometer. Both the media supported tri-lineage differentiation to an approximately equal extent. Control represents undifferentiated cells. Data are represented as mean ± SD (*n* = 3)

In SM, the cells were seen to aggregate and form a pellet-like structure when viewed under a microscope, whereas in control medium, scattered deposits of GAGs were seen. The undifferentiated hLMSCs grown in control medium and SM served as control (Fig. 3a).

The graph in Fig. 3b clearly depicts a non-significant (*p* > 0.05) difference between the extent of differentiation in both media.

### Colony forming unit (CFU) assay

**Fig. 4:**
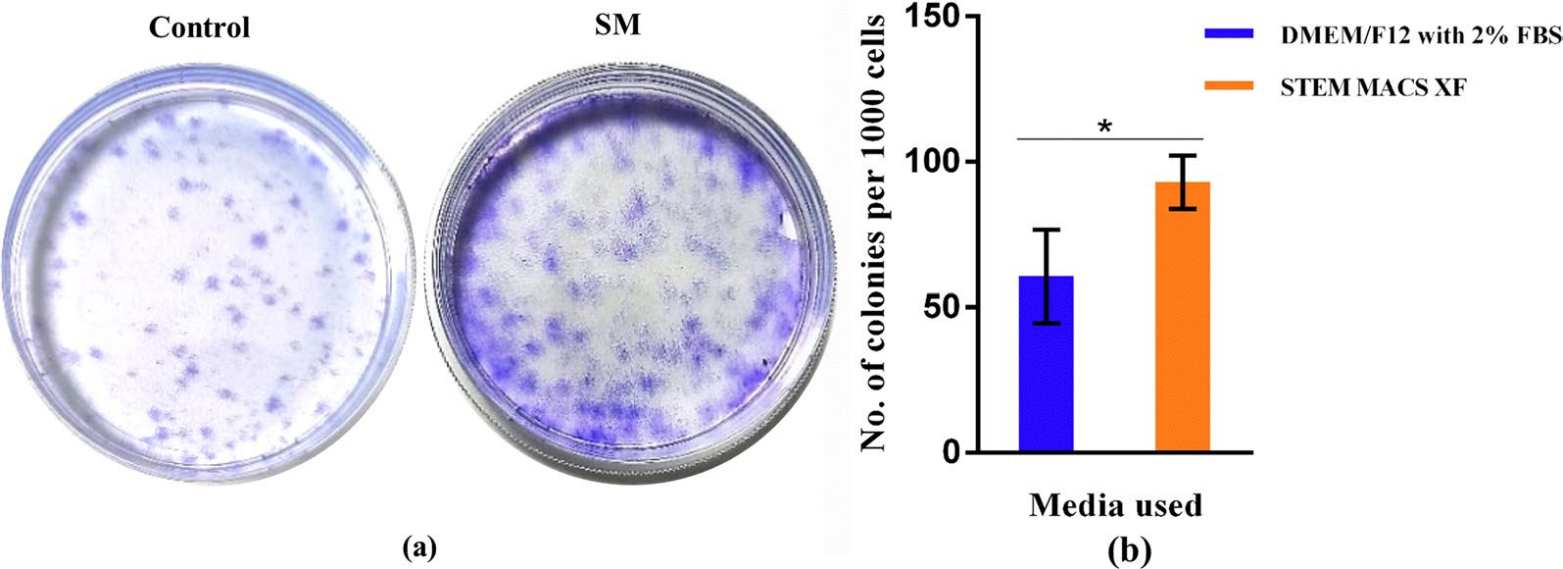
**a** Representative photograph of colony forming unit of hLMSCs: SM showed a higher number of colonies than the control medium. **b** Bar graph showing the comparison of number of colonies per 1000 cells in both the media

MSCs produce holoclones and grow in colonies when seeded at lower densities. The colonies in SM were higher and more compactly arranged, whereas, in control, they were less and scattered (Fig. 4a, b). SM had significantly more colonies than the control medium, with mean values of 93 ± 9.17 and 60.6 ± 16.01 in SM and control medium, respectively (Fig. 4c) (*p* = 0.038).

### In vitro wound-healing assay

**Fig. 5:**
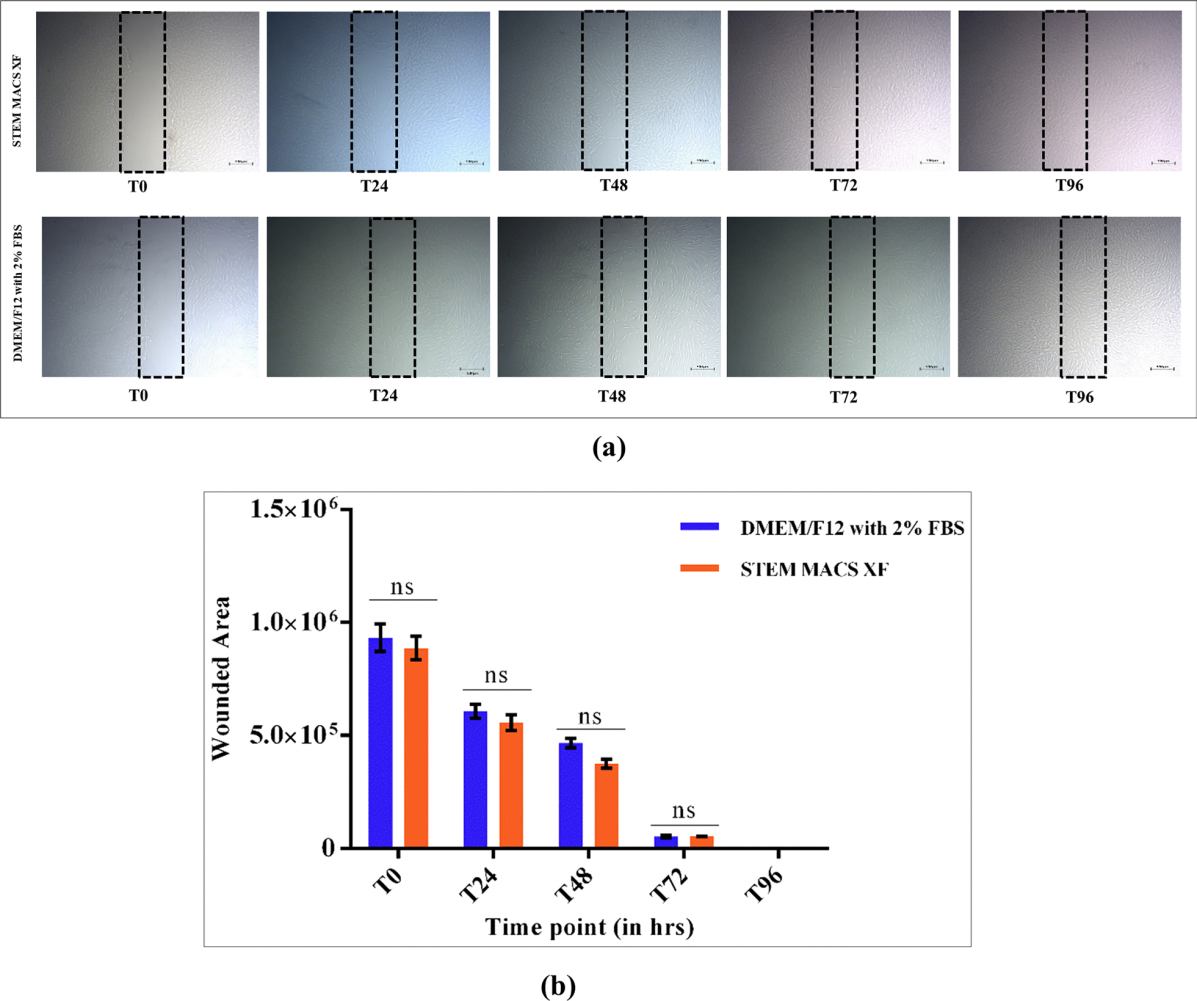
**a** Representative photograph of the wounded area of hLMSCs cultured in SM and control. **b** Bar graph showing the relative decrease in the wounded area at different time points in both media. Scale: 500 µM

hLMSCs cultured in both media displayed migration towards the wounded area without significant difference. The injured area was filled at 96 h post-wounding as shown in Fig. 5a. The average wounded area in the case of SM was found to be 886,387.5 ± 51,124.53, 556,339 ± 35,011.69, 375,298.5 ± 20,965.01, 53,199 ± 1149.756 and 0 at *T*_0_, *T*_24_, *T*_48_, *T*_72_ and *T*_96_, respectively. Similarly, in the case of the control medium, the average wounded area was 932,640 ± 61,407.98, 606,297 ± 31,133.91, 466,128.5 ± 20,034.46, 52,400.5 ± 6537.202 and 0 at T_0_, T _24_, T_48_, T_72_ and T_96_, respectively. The above data are represented on a bar graph (*p* > 0.05).

### Quantitative gene expression (qRT-PCR)

**Fig. 6:**
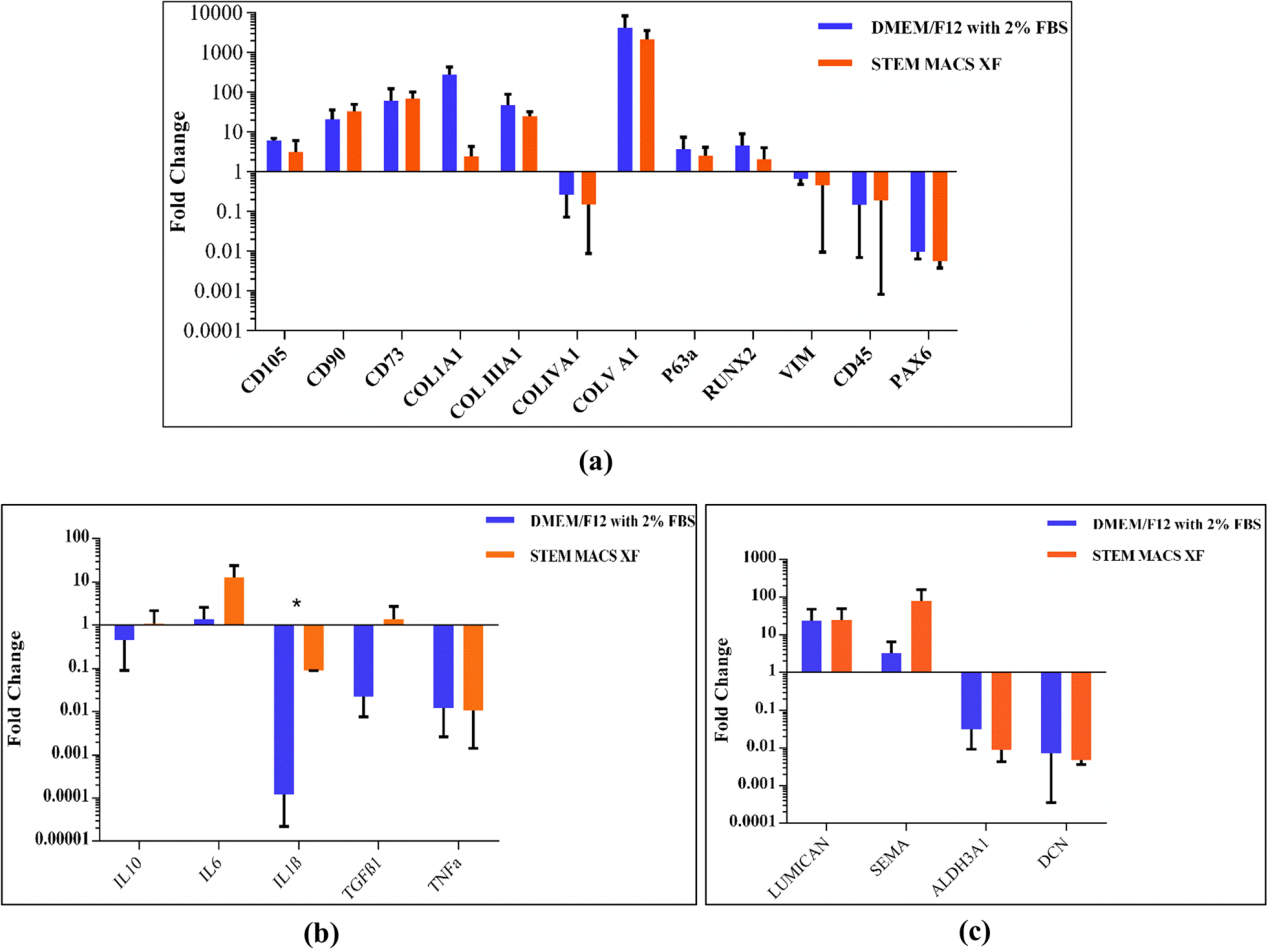
Bar graph showing log fold change of various MSC-specific genes (**a**), genes involved in inflammatory pathways (**b**), and wound healing markers (**c**). No statistically significant difference between fold change demonstrates that hLMSCs retain their genotypic and phenotyping characteristics adapting to the serum-free environment. The data are represented as mean ± SD; *n* = 3; *p < 0.5

The expression patterns of specific genes were analysed to assess the impact of xeno-free medium on various stem cell markers, wound healing and inflammatory markers. The fold change was calculated using the 2^−(ΔΔ*ct*)^ formula. All the markers showed approximately similar fold change in both the media without any significant difference except IL1β (*p* = 0.0007). The MSC markers were upregulated compared to the control except for Vimentin and PAX6 (ns). (Fig. 6a). The inflammatory markers’ expression was downregulated except IL6 compared to the native limbus (Fig. 6b). Wound healing markers like Lumican and Semaphorin were upregulated(non-significant) in both the medium, whereas Decorin and ALDH3A1 were downregulated (Fig. 6c).

## Discussion

MSCs have been used as therapeutic agents in various systemic disorders, as evident from reported clinical trials. Human corneal/limbal stroma has finger-like projections known as palisades of Vogt that house MSCs [23, 24], which promote corneal wound healing [19]. Various scientific groups have discovered diverse applications of these cells in the case of corneal scarring and haze [63] but all of them using FBS as the growth supplement to the basal media [7, 9, 10, 18]. FBS contains zoonotic antigens, which might result in cross-contamination, immune rejection and chances of bovine disease occurrence in the human population, thus compromising regulatory guidelines for transplantation. To date, MSCs have been used in various clinical trials worldwide, but they are cultured in FBS fortified medium. Hence, establishing a xeno-free method of culturing these cells has an immediate translational approach.

Some studies have cultured MSCs in low serum-containing medium [64, 65], but the idea of the complete elimination of serum would be better for therapeutic use, which led several research groups to formulate in-house xeno-free medium for MSC expansion using defined chemical compounds as supplements [66–69]. However, the safety of these in-house media haven’t been ensured, and some studies have markedly shown a difference in the growth rate of MSCs isolated from different tissues of the same organism [51, 53, 70] or from the same tissues of various organisms [71, 72]. To avoid these complications, proprietary commercially available serum-free medium like RoosterBio, Inc., MD, USA (RoosterNourish-MSC XF); Miltenyi Biotec, Germany (STEM MACS XF); Merck, USA (PLTMax Human Platelet Lysate); R&D Systems, USA (StemXVivo Serum free Human Mesenchymal Stem Cell Expansion media) for MSC growth and expansion are promising alternatives. These media contain all chemically defined supplements devoid of any zoonotic components for healthy growth of MSCs [52, 73, 74]. A study by Ghoubay et al. followed different culture conditions along with 3T3 feeder cells. They have mainly characterized the epithelial and stromal stem cells. However, our study has shown isolation of hLMSCs by looking into all the MSC-specific markers using FACS, IF and qRT PCR following the ISCT guidelines using GMP grade media. Our findings also show comparatively less culture duration of MSCs reducing the population doubling time, thereby reducing the time consumed [75].

In a similar study, Aussel C group have demonstrated the successful expansion of MSCs in serum and xeno-free medium satisfying all the required parameters similar to the results obtained in our study. There are a number of serum and xeno-free media available in the market, so different groups test MSC from different origins and using different media [76].

Another group led by Gerby S have used a single serum-free medium which has supported the growth of bone marrow mesenchymal stem cells as evident from different experiments except CFU. It would be inappropriate to say the cells are not stem cells by just using one medium to characterize. In our opinion, the Gerby S group can try other medium to characterize the cells. Other independent research groups have shown successful expansion and characterization of BM-MSCs using different other xeno-free medium [77].

As per earlier studies, optimal seeding density is required for efficient growth of MSCs, minimizing patchy growths [78]. According to a study by Abrahamsen et al., MSCs perhaps expand optimally when seeded at lower densities due to the property of contact inhibition regulated by the Wnt pathway [79]. In this study, the hLMSCs were seeded at 5000 cells/cm^2^ for all the functional assays. Maintaining a consistent PDT is vital for the translational use of MSCs. Several previous studies have claimed an equivalent doubling time of MSCs in a serum-free environment to that of serum-based medium [56, 80, 81]. The doubling time of hLMSCs in SM was maintained at 25 ± 5 h even at higher passages, while it rose significantly higher in the control medium (Fig. 1b). The cell viability percentage was also significantly higher in SM (Fig. 1e), representing better division in comparison with the control medium.

Immunofluorescence analysis of phenotypic markers revealed expression of MSC-specific surface markers (CD105^+^, CD90^+^, ABCG2^+^, ABCB5^+^, COLIV^+^, CD73^+^, and VIM^+^), negligible expression of haematopoietic markers (CD45^−^, CD34^−^, HLA-DR^−^) and fibroblastic marker (αSMA^−^) satisfying MSC criterion [2]. Earlier studies that employed serum-fortified medium to culture MSCs have reported positive expression of HLA-DR, possibly due to the presence of FGF in serum [82–84]. The depletion or complete absence of serum in culture media used in this study might have aided in minimizing HLA-DR expression in large-scale production, enhancing the therapeutic value of MSCs. In our study, the HLA-DR expression was found to be 0.88% in SM and 1.33% in the control medium (with 2% FBS) (Fig. 2b).

Differentiation of hLMSCs into all three lineages was supported by SM without any significant difference compared to the control medium (Fig. 3). Even though there was no difference in the extent of chondrogenic differentiation between the two media, the pellets were microscopically different in size (Fig. 3 middle panel). In case of SM, the pellet was more prominent, whereas the GAG deposits were scattered throughout the plate in the control medium. Further, the colony-forming ability was significantly higher in SM compared to the control medium (Fig. 4; *p* = 0.038). In vitro wound healing assay, a property well exhibited by MSCs, was also retained in the serum-free formulation. In fact, SM demonstrated better healing potential of hLMSCs than the control medium, due to comparatively lower population doubling time (Fig. 5a).

The gene expression pattern was observed to be similar in both media, as evident from the calculated fold change in qRT PCR (Fig. 6). Various stem cell markers were over-expressed in both media as compared to the native limbus, except Collagen IV, Vimentin and PAX6. As collagen IV is mainly present in Descemet’s membrane, the expression is supposed to be higher in native tissue [85, 86]. Similarly, concerning PAX6, which is omnipresent in both corneal and limbal epithelia relative to the stroma, its decreased expression in hLMSCs is self-explanatory [87].

The culture of corneal/limbal stromal cells in the serum-free medium has been reported in previous studies, but those media weren’t adequately characterized. Some studies used human platelet lysate, which again has lot-to-lot variation [88]. In this study, we demonstrated the growth and expansion of hLMSCs in vitro in serum-free conditions using a commercially available xeno-free medium, SM. SM was chosen as it has been successfully used in the expansion of BM-MSC [4] and was readily available. As SM hasn’t been used in the culture of limbal stromal cells, we tried to explore its potential for hLMSCs. This medium is manufactured in a GMP-compliant facility, which is an added advantage for therapeutic use.

Undoubtedly, we acknowledge certain limitations of this study, like characterization and optimization of only P3 hLMSCs and usage of only one serum-free medium. However, this study successfully addresses the aim of expanding P3 hLMSCs in a serum-free environment.

Optimization of hLMSCs in SM at higher passages and to study the safety and toxicity of these cells in animal models needs to be further explored.

## Conclusion

The findings of this study suggest that the phenotypic and functional property of hLMSCs is retained in serum-free environment. Further, their ability for wound closure and multi-lineage differentiation also remains unaltered. This indicates that the serum-free medium not only supports but also enhances its characteristic features, in addition to overcoming regulatory and ethical constraints.

## Supplementary Information

**Additional file 1.** Immunofluorescence analysis of epithelial markers in Human Corneal Epithelial (HCE) cell line and hLMSCs.

## Data Availability

All the data are available with the corresponding author upon request.

## Abbreviations

hLMSCs: Human limbus-derived stromal/mesenchymal stem cells
MSC: Mesenchymal stem cells
LSCD: Limbal stem cell deficiency
SM: STEM MACS XF
FBS: Foetal bovine serum
PDT: Population doubling time
BM: Bone marrow
UC: Umbilical cord
AD: Adipose tissue
CSSC: Corneal stromal stem cells
RIEB: Ramayamma International Eye Bank
PBS: Phosphate-buffered saline
IF: Immunofluorescence
BSA: Bovine serum albumin
CPD: Cumulative population doublings
GAG: Glycosaminoglycans
EGF: Epidermal growth factor
DMEM: Dulbecco’s modified eagle medium
CD: Cluster of differentiation
T: Time
P3: Passage 3
