# Supplementary material for: Expansion and characterization of human limbus-derived stromal/mesenchymal stem cells in xeno-free medium for therapeutic applications
Source: Stem Cell Res Ther. 2023 Apr 15;14:89. doi: 10.1186/s13287-023-03299-3 (PMC10105964; doi:10.1186/s13287-023-03299-3)
Supplement: Supplementary file 1 — Additional file 1. Immunofluorescence analysis of epithelial markers in Human Corneal Epithelial (HCE) cell line and hLMSCs. [file 13287_2023_3299_MOESM1_ESM.docx]

**Additional file 1**

**Supplementary Fig S1:** Immunofluorescence image showing the expression of typical epithelial markers CK3/2P, CK14 and CK15 in Human Corneal Epithelial (HCE) cell line, whereas no expression in P3 hLMSCs. ***Blue: DAPI; Scale: 50µm; Magnification 20X.***

**CK3/2P**

**CK14**

**CK15**

**STEM MACS XF**


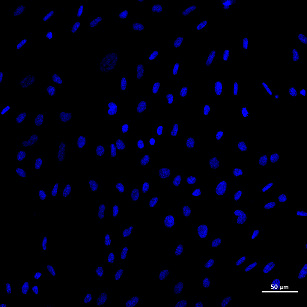

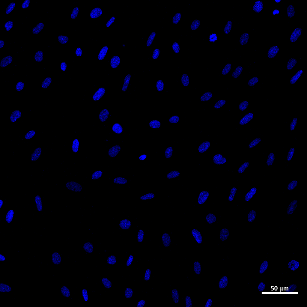

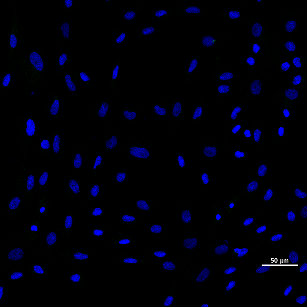


**DMEM /F12 with 2% FBS**


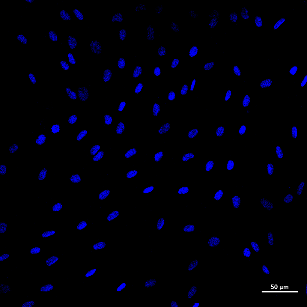

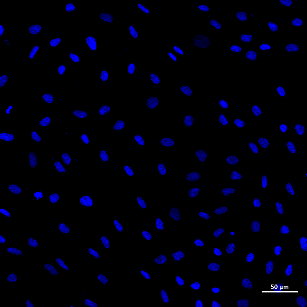

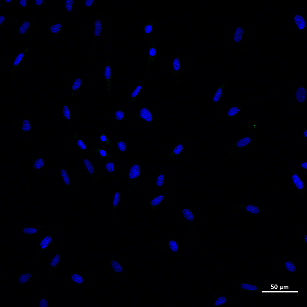

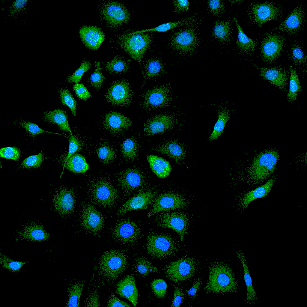

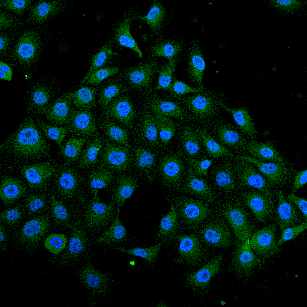

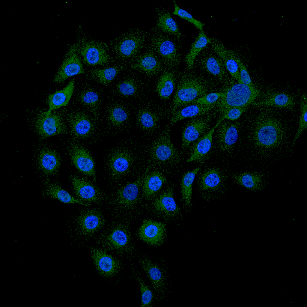


**HCE**

**(Control)**

**hLMSCs**
